# Optical absorbance profilometry for tracking time-resolved particle redistribution in high volume fraction colloidal droplets

**DOI:** 10.1038/s41598-024-51250-0

**Published:** 2024-01-05

**Authors:** Sheila J. Bhatt, Alexander F. Routh

**Affiliations:** Department of Chemical Engineering and Biotechnology, Institute for Environmental and Energy Flows, University of Cambridge, Phillipa Fawcett Drive, Cambridge, CB3 0AS UK

**Keywords:** Chemical engineering, Fluid dynamics

## Abstract

The distribution of components within colloidal suspensions is important in many complex biological and industrial fluids. A convenient method of measuring such distributions in low-volume-fraction suspensions is that of optical absorbance. Here we introduce a time-dependent validity criterion allowing extended use of optical absorbance to track colloidal distribution in high volume fraction suspensions. We define our validity criterion and show its use on a range of volume fractions from 15 to 55%, and also on larger micron sized particles, common for biological cells. Within the validity criterion, we establish the evaporative time duration in which the material’s intrinsic coefficient of extinction can be treated as constant. This method enables rapid, low-cost, time-based study of the advective flow of suspended particulates, enabling advection to be straightforwardly measured from digital imaging. The residue profile predicted using our method in two test systems is compared with conventional laser profilometry measurements of the final evaporated residue, with good agreement at most radial positions.

## Introduction

The redistribution of particulates in colloidal suspensions is an important measurement in a number of biological^1^, industrial^2^ and environmental^3^ fields, such as coatings, atmospheric monitoring, and cell count. The redistribution in droplets has been the subject of studies that attempt to predict the final dried morphology via simulations based on lubrication theory^4–6^. Particles in sessile droplets redistribute during evaporation due to internal flows^7^ as in the frequently reported “coffee-ring”^8^ residue formation, where particles accumulate at the droplet edge. The resulting residue has been investigated using many techniques^9^. Experimental measurements of colloidal distribution profiles include techniques based on NMR (nuclear magnetic resonance)^10^ to directly track such flows. However, these are expensive and require greater interpretation of results than direct observation. Interferometric techniques^11^ and fluorescent tracers^12^ provide accuracy at the cost of experimental complexity. A simple experimental means of tracking the evolution of topological height structures in colloidal drops during evaporation would be of value.

The Beer-Lambert law, expressed in Eq. (1), is based on measuring the attenuation of light transmitted through a sample. Though light spectrophotometry is closely associated with Eq. (1) and is a common technique, it does not seek to track the evolution of particulate distribution within a sample: it measures the average volume fraction of a dilute sample. The drawback is that concentrated samples must be diluted to ensure accurate average volume fraction measurements in a cuvette geometry.

1 $${A =-log}_{10}\left(\frac{I}{{I}_{0}}\right)=\varepsilon . \phi . h$$

here $A$ is the absorbance, *I* is the transmitted light intensity, *I*_o_ is the unimpeded intensity of the incident light, ε is the extinction coefficient, ϕ is the volume fraction of the sample, and $h$ is the path length of light passing through the suspension. Equation (1) is also conventionally used with a sample flowing past a sensor to obtain an average turbidity measurement. Raw transmittance has been used for optical volume fraction measurements^13^ without the use of Eq. (1). In other work absolute emission-intensity has been used in excitation fluorescence (tracer) measurements^14^.

Equation (1) is generally considered to be valid, in absolute terms, only for low values of particle volume fraction. Recent studies have reviewed the use of Eq. (1), including in complex biological systems^1^ and other scattering samples^15^, concluding that a variety of case-based modifications may permit extended use. We show that in small droplet samples where gravity can be neglected, Eq. (1) is practical for relative measurements, enabling tracking of the advection of high-volume fractions of suspended solute provided the extinction coefficient remains constant,

As noted above, optical techniques are known to suffer from reduced accuracy when applied to high volume-fraction suspensions^3^. Here we show a means of validating a period during which the extinction-coefficient for absorption is constant for such suspensions. We show that this criterion coupled with diffuse illumination can provide a means of probing redistribution of the larger colloidal lengthscale of biological cells. We illustrate this with 6 μm, diameter, polystyrene spheres^16^ in neutrally buoyant aqueous carrier-fluid up to volume-fractions of 55%, as well as with similarly sized red blood cells in a neutral carrier-fluid.

Significant colour-density in some samples can also result in increased absorbance or scattering of certain wavelengths^17^, meaning that wavelength-selection and channel-splitting prior to analysis may be required. Recent work based on differential optical absorbance between colour-channels noted a difficulty in applicability to larger particles, and required both added dye and a fixed thin film pathlength in order to track distribution in drying films^18^. Here we show that meaningful redistribution can be tracked without such restrictions or additions. We show good results corroborated by profilometry of the solute residue.

## Materials and methods

The technique presented here relies on Eq. (1) to interpret absorbance of a diffuse white light source directed perpendicular to the sample plane. The sample geometry is that of a sessile pinned droplet with a shape governed by surface tension. We track the change from an initially homogeneous solute distribution as a function of time and position.

**Figure 1:**
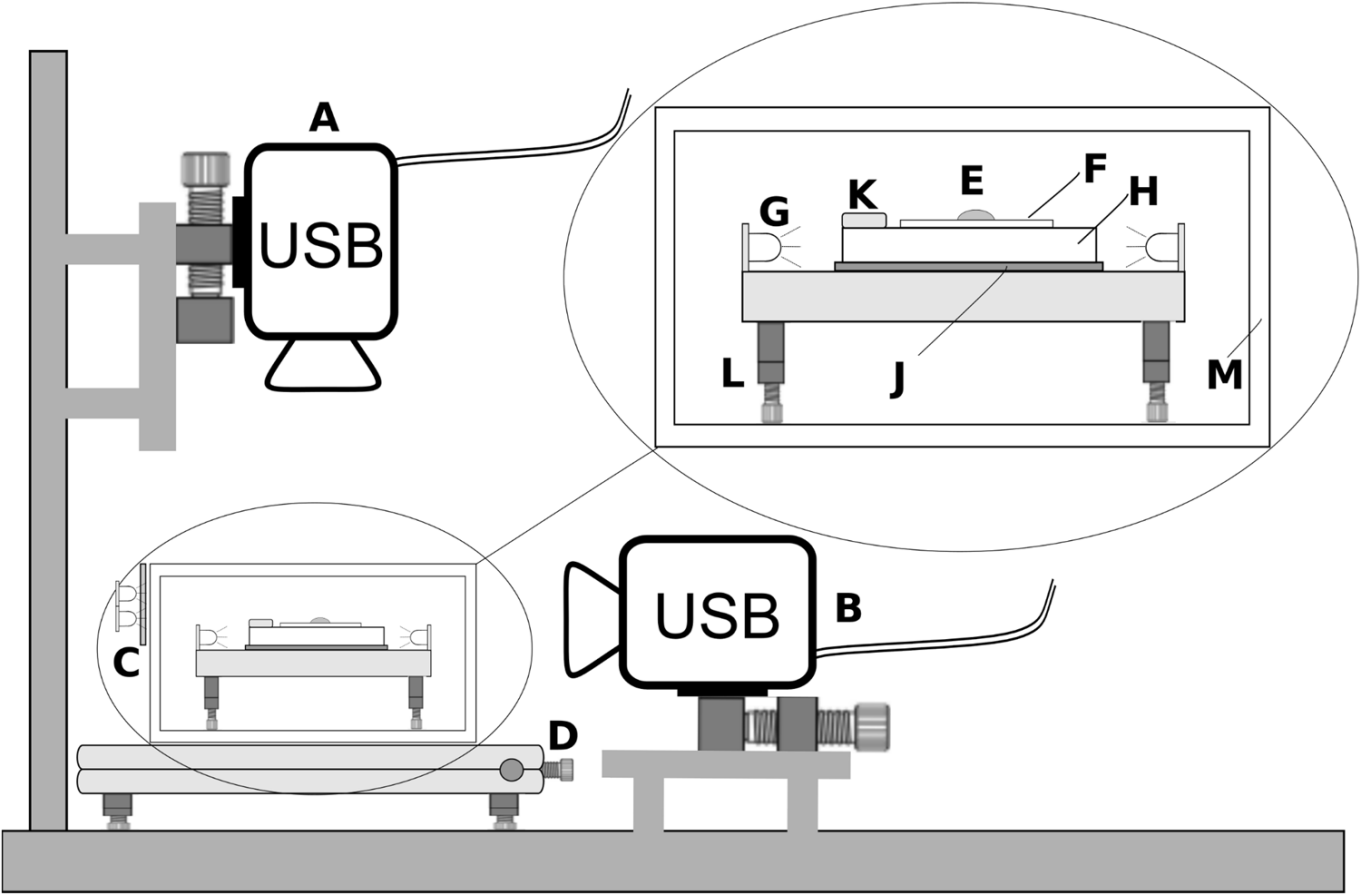
Multi-camera apparatus: (**A**) Top camera on vertical focus-rail (DaHeng 12 Mpx MER1200 with 1/1.7" IMX226 sensor + Canon MP-E65 macro lens). (**B**) Side-camera on horizontal focus-rail (DaHeng 6 Mpx MER630 with 1/1.8" IMX178 sensor + Fujian GDS-35 macro-lens) (**C**) 12v LED low intensity lighting and diffuser illuminating side silhouette capture for Camera-B, (**D**) XY sample-positioning controls INSET: Sample-Stage. (**E**) sample, (**F**) microscope slide, (**G**) 12v LED light sources for edge-lit panel illuminating the sample from beneath for absorbance capture by Camera-A, (**H**): translucent acrylic edge-lit platform, (**J**) thin-film resistive heater, (**K**) wireless temperature and humidity sensor, (**L**) levelling feet, (**M**) transparent acrylic environment box.

The apparatus constructed for this technique is shown in Fig. 1. Two USB3 digital cameras provide, A-top-down and B-side-on images. Microscope slides on which to deposit samples for observation, are mounted on an acrylic underlit sample-stage, energised by a peripheral array of inward-facing LEDs (yielding diffuse illumination beneath the sample). A low height and low intensity vertical LED panel shuttered to align with the side-view camera B illuminates the far side of the sample for silhouette imaging, allowing absolute height measurements and contact-angle analysis. Power to the LED panels is controlled via a variable laboratory PSU (Evertek KPS1505DF) in constant-current mode. The underlit sample-stage is mounted on an X–Y platform controlled by twin vernier adjusters, enabling accurate sample positioning. The platform is levelled using four independent screw-feet and a levelling bulb. The sample is protected from aberrant airflow by an environmental box mounted on the adjustable stage. Temperature and humidity are recorded using a wireless sensor [(WirelessTag Pro, accuracy ± 0.38 °C) with data recorded into an online TSDB (Time Series Database)^19^]. They are an indication of the evaporation environment and are not a direct measure the droplet’s temperature. An adjustable dosing guide is mounted above the sample-stage to enable accurate droplet-placement at the combined focal volume of both cameras via a 0.1–10 μL autopipette (SciPette YM209AL0010969), thus allowing synchronised image-capture from the moment of droplet-deposition. The dosing guide position can be adjusted in three dimensions.

Custom Python software was used to control the cameras and LEDS and allowed the capture of time-sequence images at variable rates appropriate to each sample. The software also ensures each image is overlaid with a time/date-stamp and descriptive text.

Calibration of the side camera was carried out by imaging a microscope slide of known thickness, to relate the absolute dimension to a known number of pixels. Contributions to absorbance measurements from carrier-fluid were assessed by evaporating a droplet of carrier-fluid with no solute present. Droplet curvature might imply lensing and calibration experiments were conducted without solute. Clear droplets are the worst-case scenario in which to assess any potential lensing. The results show lensing effects to be negligible.

## Experimental procedure

Suspensions of 6 µm spheres at varying volume fractions were prepared from monodispersed polystyrene spheres (Unibead 6-1-0600, density 1060 kg m^−3^, Chromatech Research), using a bench-top micro-balance (On Balance CT-250), and an automatic 0.1–10 µL pipette (SciPette YM209AL0010969). The polystyrene spheres were suspended in a density-matched H_2_O/D_2_O carrier-fluid composed of equal parts of H_2_O and D_2_O (Aldrich 151882). Prior to use, samples were left to equilibrate for 30 min and then gently agitated for 60 s using a bench-top agitator (MS1 Minishaker). Density matching ensured that settling times were longer than the valid experimental periods. Blood samples were obtained from healthy donors with informed consent under ethical approval RES/CD/2021/22. Samples were prepared by centrifugation of donated blood, followed by washing with PBS and resuspension in neutral buffered carrier-fluid as above.

The X–Y stage and lens were adjusted to centre the coincident focal volume of both cameras at the dosing-guide deposition-point of the pipette tip. For each experiment, a new microscope slide was mounted on the imaging stage and allowed to reach ambient temperature. Humidity and temperature in the environment box were noted. A 2 μL droplet was deposited on the substrate at the combined focal volume using the automatic pipette in the dosing guide, and synchronised capture from both cameras was started. Recording continued until the images showed no further feature or intensity changes.

Calibration of the optical uniformity of the apparatus was carried out on a clean glass microscope slide with no droplet present. The results showed that detection of incident light was uniform to within ± 1%.

Calibration without particulates showed absorbance by the carrier-fluid typically accounted for < 0.1% of the measured absorbance, so for this small droplet geometry, variations in the optical path length $h$ through the carrier fluid were ignored. These results demonstrate that carrier-fluid contributions and background-intensity variations can both be neglected when optical absorbance is measured within the validity period of constant extinction coefficient.

### Analysis

In the following, we assume low Reynolds-number flow in the droplet. We assume rapid diffusion, ensuring vertical equilibrium in a neutrally buoyant carrier fluid. We deem an *end-of-advection time* to be when there is insufficient carrier fluid to significantly redistribute the solute in any region of the droplet. After *end-of-advection*, we identify a ‘modified extinction coefficient’ during de-wetting and eventual desiccation.

To extract quantitative data, a number of numerical image-analysis techniques were utilised. The images were split into RGB colour-channels and the channel with optimal transmission selected. Each of the selected images was then analysed using ImageJ’s ‘concentric circles’ plugin to obtain the circumferentially-averaged intensity for 100 concentric radial positions. These were equally spaced to cover the droplet, starting 5 pixels from the centre to just beyond the visible rim. A circle beyond the sample rim was measured to yield the average intensity of the incident light ${I}_{0}$.

Measuring the central circle average absorbance at time *t* = 0, and scaling the droplet pathlength ${h}_{0}$ to have a value of unity, we use Eq. (1) to calculate the combined factor of ε. ${\phi }_{0}$ for the initially-homogeneous suspension at time *t*_*o*_. Equation (1) is then applied using this factor to each of the radially averaged absorbance values.

This then allows the ratio of absorbance $A/{A}_{0}$ to be used to track advection over time, as follows:

For time *t* = 0, Eq. (1) yields ${A}_{0}=-{log}_{10}\left(\frac{I}{{I}_{0}}\right)={\varepsilon }_{0} {. \phi }_{0}{ . h}_{0}$

Then at some later time t, $A=\varepsilon . \phi . h$. Dividing these expressions and scaling ${h}_{0}$ to 1 yields

2 $$\frac{A}{{A}_{0}}= \frac{\varepsilon }{{\varepsilon }_{0}} . \frac{ \phi }{{ { \phi }_{0}}} .h$$

which, if the extinction coefficient remains constant (from *t* = 0 until *t*), gives $\frac{A}{{A}_{0}}= \frac{ \phi }{{ { \phi }_{0}}} .h$

For constant extinction coefficient *ε*, relative changes in absorbance between radii therefore yield the distribution of ϕ $h$ at each radial position. We further note that at the end of advection, the packed area has reached steady state with an assumed volume fraction, ${\phi }_{m}$ of 0.64 (for random close packed spheres). From this, we can obtain the height profile of the absorbing particles.

3 $$h= \frac{A}{{A}_{0}} .\frac{{ \phi }_{0}}{{{ \phi }_{m}}}$$

This analysis applies at each radial position in each image in the time-sequence. We observe the accumulation of packed particles at the droplet edge. The absorbance value in this region where the particle volume fraction is constant ${\phi }_{m}$ therefore corresponds to the droplet height neglecting the carrier-fluid.

To identify the validity for Eq. (1), we consider the total measured absorbance. No solute mass is lost during evaporation, and ε is considered constant, so if the absorbance due to carrier-fluid can be neglected, the integral of total absorbance should remain constant over time***.***

The continuity expression is

$$\underset{r=0}{\overset{r={R}_{max}}{\int }}2\pi .r. \phi . h.dr = constant$$

and hence we calculate

4 $$\underset{r=0}{\overset{r={R}_{max}}{\int }}A(r) .r.dr = constant$$

**Figure 2:**
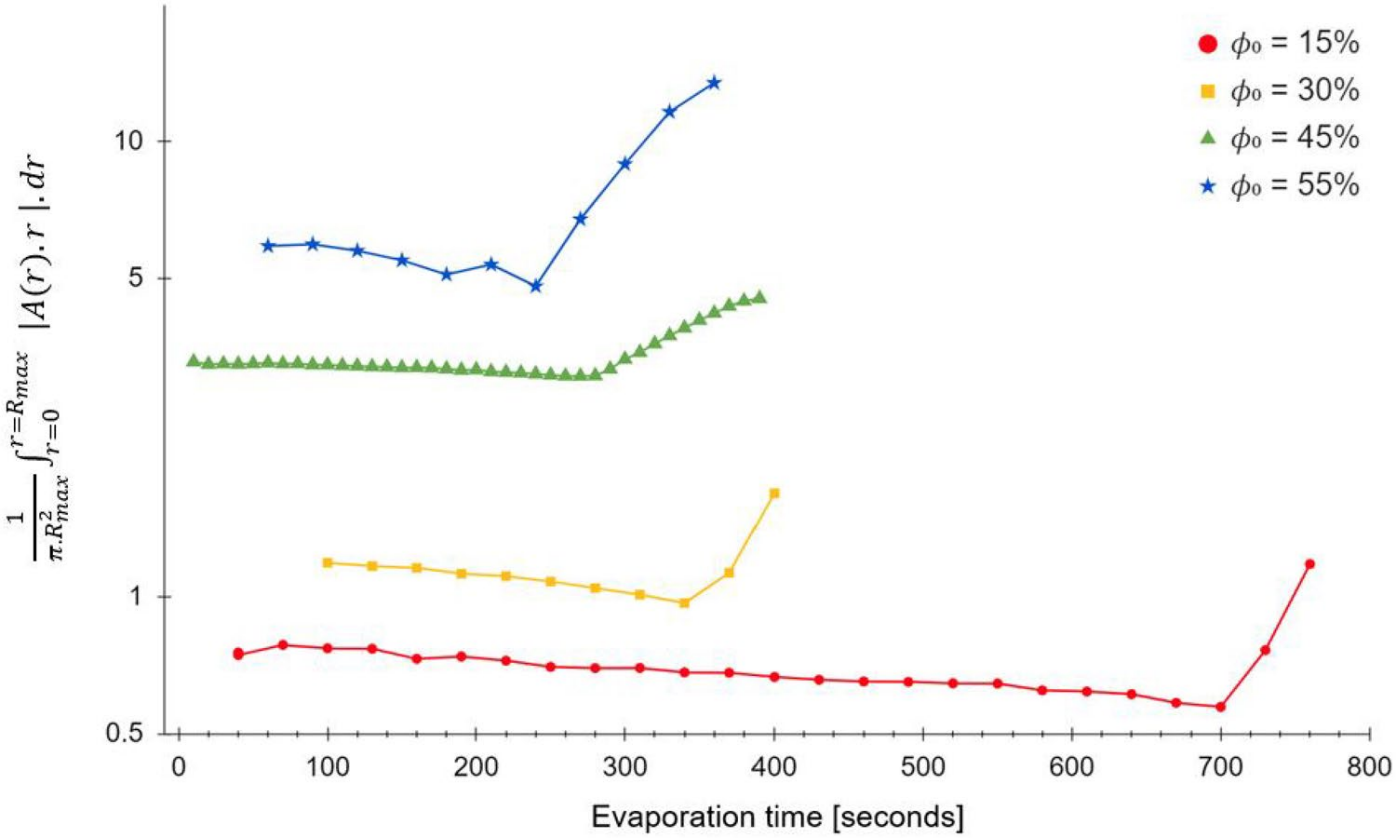
$\frac{1}{\pi .{R}_{max}^{2}}{\int }_{r=0}^{r={R}_{max}}\left|A(r).r\right|.dr$ as a function of time, for differing initial volume fractions ${\upphi }_{0}$ of 6 μm polystyrene spheres in density-matched H_2_O/D_2_O, exhibiting a distinct change in gradient at critical times t_ex_ . Normalisation by the area of the droplet shows the data to be monotonic in initial volume fraction.

A plot of this integral as a function of time is shown in Fig. 2, which shows a slight early time decline followed by a rapid increase at a specific time, which we call *t*_*ex*_. This corresponds to the point of air invasion into the film, and hence a change in the extinction coefficient.

The principal sources of uncertainty in this arise from errors in measuring position, volume fraction and absorbance / intensity values. These are assessed as follows:

- Positional: systematic error of ± 2 pixels in camera-resolution, typically < 0.01% and random error from blurred feature identification leads to errors estimated at ± 4% in positions, and ± 8% in velocity.
- Volume fraction: ± 2% mass error from a digital micro-balance, and ± 3% error in pipette volumes.
- Intensity: systematic errors in averaged pixel intensity from concentric circles vary from ± 2% for minimum radius, falling to ± 0.01% for maximum radius, and camera error of 1/255 (± 0.4%), and random error due to deviations in droplet rim circularity imply measurement-circles near the edge may intersect background; estimated worst case error ± 7% within 20 pixels of droplet rim, zero elsewhere. The Intensity-ratio doubles errors for each absorbance value, then scales by 0.434 due to log_10_, giving total errors in derived absorbance of ± 2.8% (rim) to ± 9%(centre), depending on radial position.

It is noted that in the case of significant carrier-fluid absorbance, this analysis could be modified to adjust $h$ accordingly, from an image sequence and volume fraction profiles.

## Results and discussion

Figure 2 shows the integral of absorbance over time for four initial volume fractions ranging from 15 to 55%, with an early gradual decline in the integral of absorbance, of all samples. The time at which the graph showed an appreciable positive change in gradient was taken as defining a critical time *t*_*ex*_ . Unsurprisingly, it is seen that the value of *t*_*ex*_ drops with higher initial particle loadings. The measured value of *t*_*ex*_ was then used to scale time.

The initial decline is likely due to loss of solvent, and the later increase likely due to air ingress leading to enhanced scattering. In what follows, we only consider time less than *t*_*ex*_ for determining droplet profiles.

**Figure 3:**
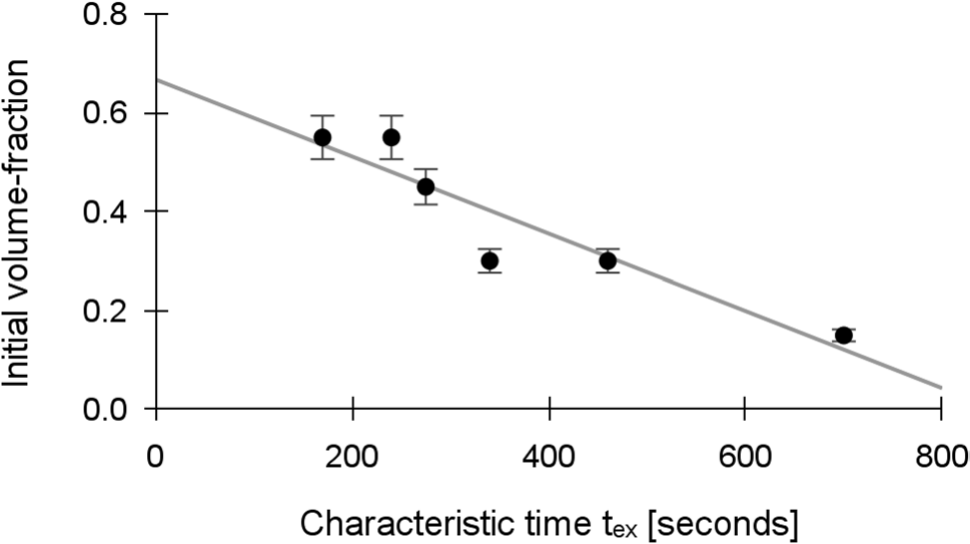
Characteristic time *t*_*ex*_ as a function of initial volume-fraction ${\phi }_{0}$ of solute, for 6 μm polystyrene spheres in density-matched H_2_O/D_2_O.

Figure 3 shows the variation of *t*_*ex*_ with initial volume fraction ${\phi }_{0}$ .

The intercept on the vertical axis of this plot gives an experimentally-derived estimate for the maximum possible particle volume fraction in this system, ${\phi }_{m}$ = 0.67, close to the ${\phi }_{m}$ = 0.64 value based on an assumption of spherical random close-packing. Figure 3 may therefore be used to obtain initial-volume fraction values directly from the observed value of *t*_*ex* ._

**Figure 4:**
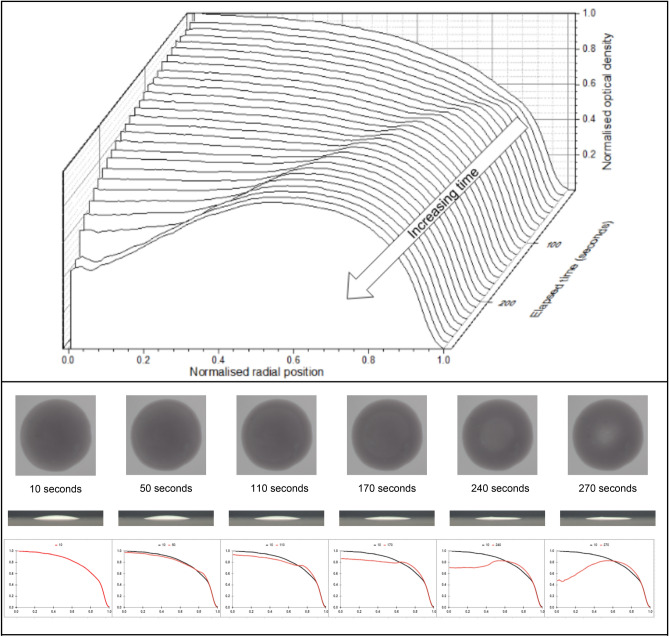
Time-sequence of images and absorbance-profiles of polystyrene 6 μm spheres in density-matched H_2_O/D_2_O initial homogeneous volume fraction ${\phi }_{0}$ = 45%; *T* = 23 °C *RH* = 35%, substrate: glass.

Figure 4 shows the evolution of the radial profile of optical absorbance as a set of contours at ten-second intervals as a 3D array with time on the third axis.

The initial particle distribution is homogeneous, confirmed by the initial absorbance profile matching the side camera images. Subsequent absorbance profiles show gradual changes due to the redistribution of particles under advective flows. We note a number of key times and observations:

1. The absorbance profile develops a peak near the rim at early-time, which grows inwards with time
2. The absorbance contour at the centre declines steadily until late-time, where it drops significantly in the final time intervals.
3. Note that after deposition, it takes around 1 s to establish the equilibrium contact angle

The distribution contours suggest solute being advected from the central regions to the rim where it packs. We explain this as follows: The droplet is initially a spherical cap with a homogeneous distribution of particles. Evaporation may be enhanced at the rim^20^, but this is not necessary for the formation of a coffee-ring^21^. The reduced height at the droplet edge causes particles in this region to consolidate into close packing. Continued evaporation of carrier-fluid from this consolidated region causes a flux from the bulk towards the rim, which carries particles and propagates the front of close packed particles towards the droplet centre. Flow of solute ceases when there is insufficient remaining carrier-fluid to support advection. This results in the well-known ‘coffee-ring’ residue-pattern.

The optical technique produces a means of monitoring both the full plain of the droplet and the solute distribution. We note that the plotted profiles are tracking relative solute distribution, not the droplet surface.

**Figure 5:**
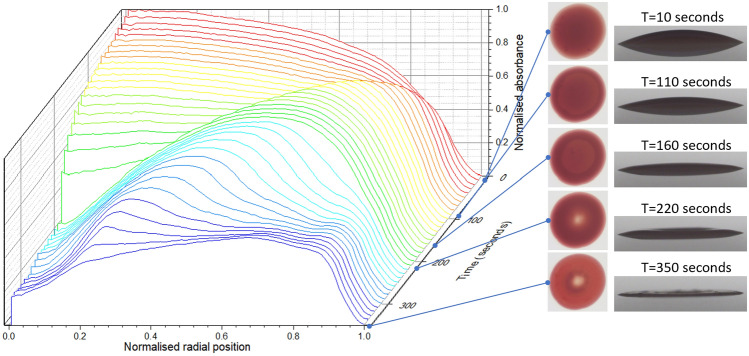
Time-sequence of images and absorbance-profiles of red blood cells resuspended in carrier-fluid. Initial volume fraction ${\phi }_{0}$ = 38%; Environmental: *T* = 22.3 °C, *RH* = 23%, substrate: polysine-coated glass. Note the biological fluid dries in a markedly different fashion from the polystyrene dispersion.

Figure 5 illustrates the application of the technique to another colloidal system: that of red blood cells re-suspended in a neutral carrier-fluid. The growth and movement of a congestion-front can be clearly observed, followed by the formation of a coffee-ring deposit from the residue.

**Figure 6:**
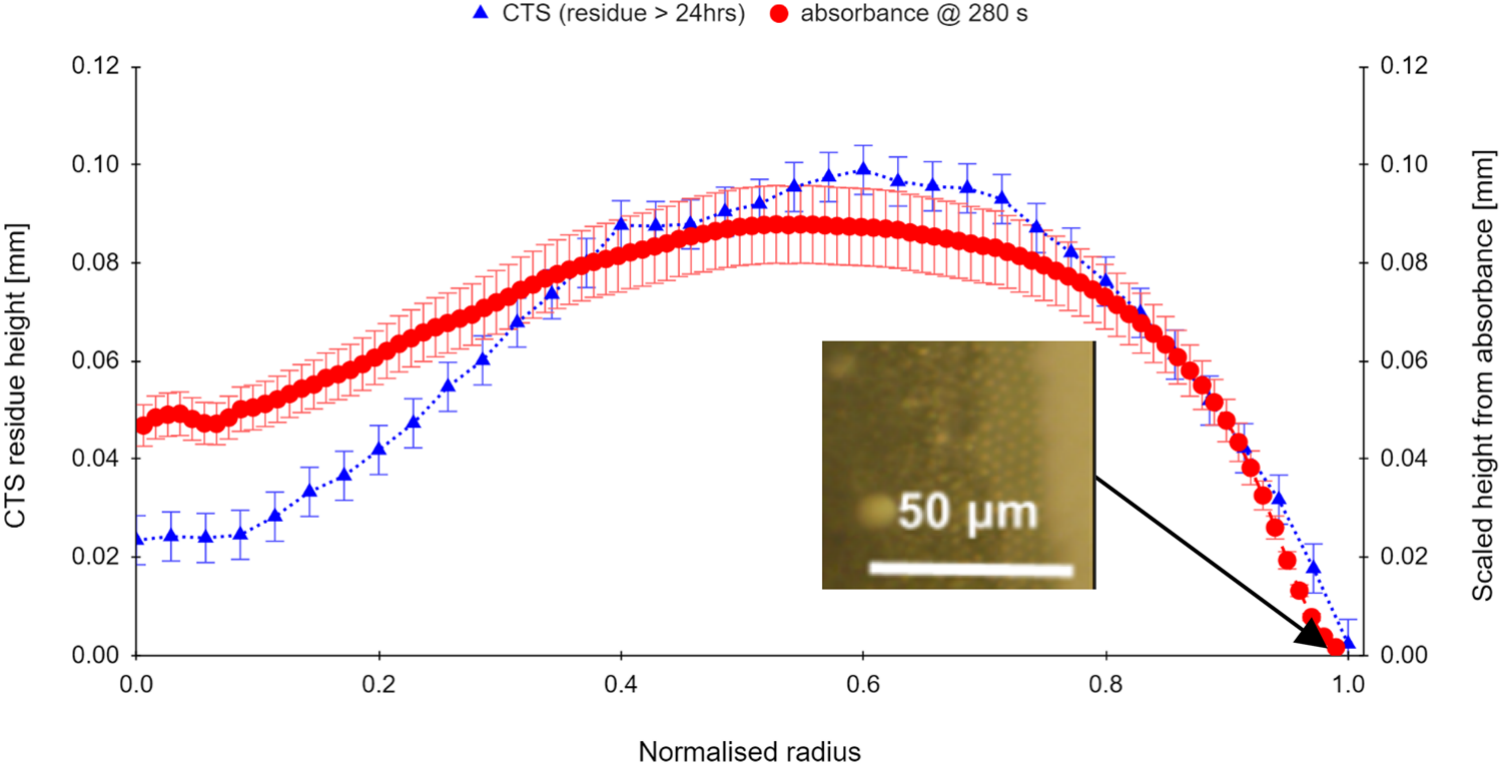
Comparison of the predicted residue height profile with final CTS measured surface profile, volume fraction ${\phi }_{0}$ = 45%; *T* = 23 °C; *RH* = 35%, substrate: glass. Predicted height profile is scaled to mm using the side-camera image. Inset shows close-packed well-ordered monolayer of particles at the rim, transitioning to random packing moving inward from the rim. CTS measured at > 24 h.

In Fig. 6 a dried residue profile was measured using a laser confocal thickness scanner profilometer (Micro-Epsilon confocal thickness sensor CTS-DT2405/3) and compared with the final absorbance profile at 280 s, converted to millimetres via absolute height-measurement from a calibrated side-view image of the initial droplet. Profiles obtained from both instruments are in broad agreement.

Thickness measurement errors for the CTS instrument are estimated at ± 5 μm and can suffer from noise, especially for sample-thickness below 150 μm (which is the minimum rated height). The error estimate falls to ± 1.5 μm above 150 μm.

The final absorbance profile contour compared here in Fig. 6 shows good general agreement with the confocal thickness scan (CTS) of the desiccated residue. However, we note that the absorbance measurements converted to height read higher than the CTS for the droplet centre, and slightly lower for the ‘crown’ of the peak region. Discrepancies may be due to fewer points being averaged in the inner smaller concentric circles, or air ingress leading to enhanced scattering.

The higher central absorbance may be linked to the late-time feature noted in many studies colloquially as *rush-hour*^22^; where the increase in the outward radial velocity of the last of the carrier-fluid close to the time t_ex._rapidly drains the central region. As the central region cannot be replenished at this point, this leads to a sudden desaturation of the residue in the central region.

**Figure 7:**
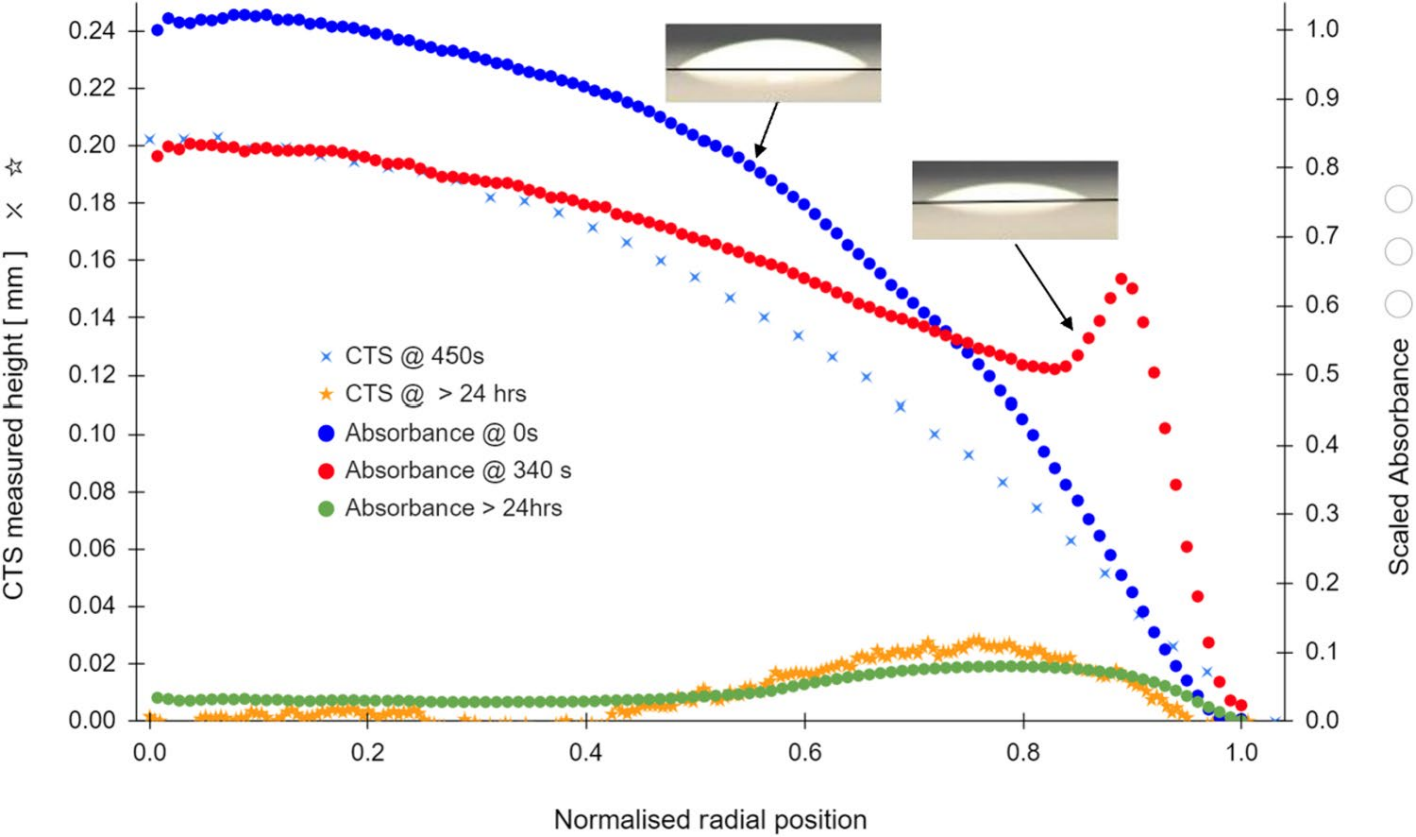
Comparative CTS (left axis) and absorbance (right axis) profiles for a partially-evaporated droplet of polystyrene in H_2_O/D_2_O volume fraction ${\phi }_{0}$ = 15%; *T* = 23 °C, *RH* = 33%, Note the above shows that the absorbance technique is able to track advection of the suspended solute during evaporation which the CTS technique cannot resolve. The absorbance profile at time t_o_ , shows an initially homogeneous distribution of solute that maps closely to the droplet topology. Since the absorbance tracks the solute advection, after 340 s the ‘peaked contour’ represents the redistribution of the solute. The peak shown in the 340 s absorbance contour does not represent a physical protrusion above the initial droplet surface, but a local increase in solute volume fraction. For completeness the lower insert shows the CTS and absorbance measurements of the residue after evaporation.

In Fig. 7, a sample was monitored using the absorbance technique for an initial period of 340 s, during which the start of redistribution of the solute towards the droplet rim is observed. After 340 s the sample was transferred to the CTS and a height profile measurement obtained at a time of 450 s.

The CTS profile shows a smooth spherical cap droplet profile, with no indication of solute redistribution, whereas the absorbance contour at 340 s shows advection of material from the central area towards the rim, as the coffee-ring is established. We note that the droplet at this juncture has a smooth wet free surface confirmed by side view images (see Fig. 7 upper right insert). However the volume fraction $\phi$ of particulates in this thin rim (after 340 s) has already risen substantially above the homogeneous ${\phi }_{0}$ which was the value at this radius, at time *t* = *0*.

Comparison of the two techniques confirms that the absorbance technique is measuring local redistribution of particulates. It is measuring the local value of $\phi . h$ rather than $h$. Even in the shortest optical pathlengths, the absorbance contour gives a good indication of the proportion of solute that will be found close to the rim, which for these samples forms the classic ‘coffee-ring’.

**Figure 8:**
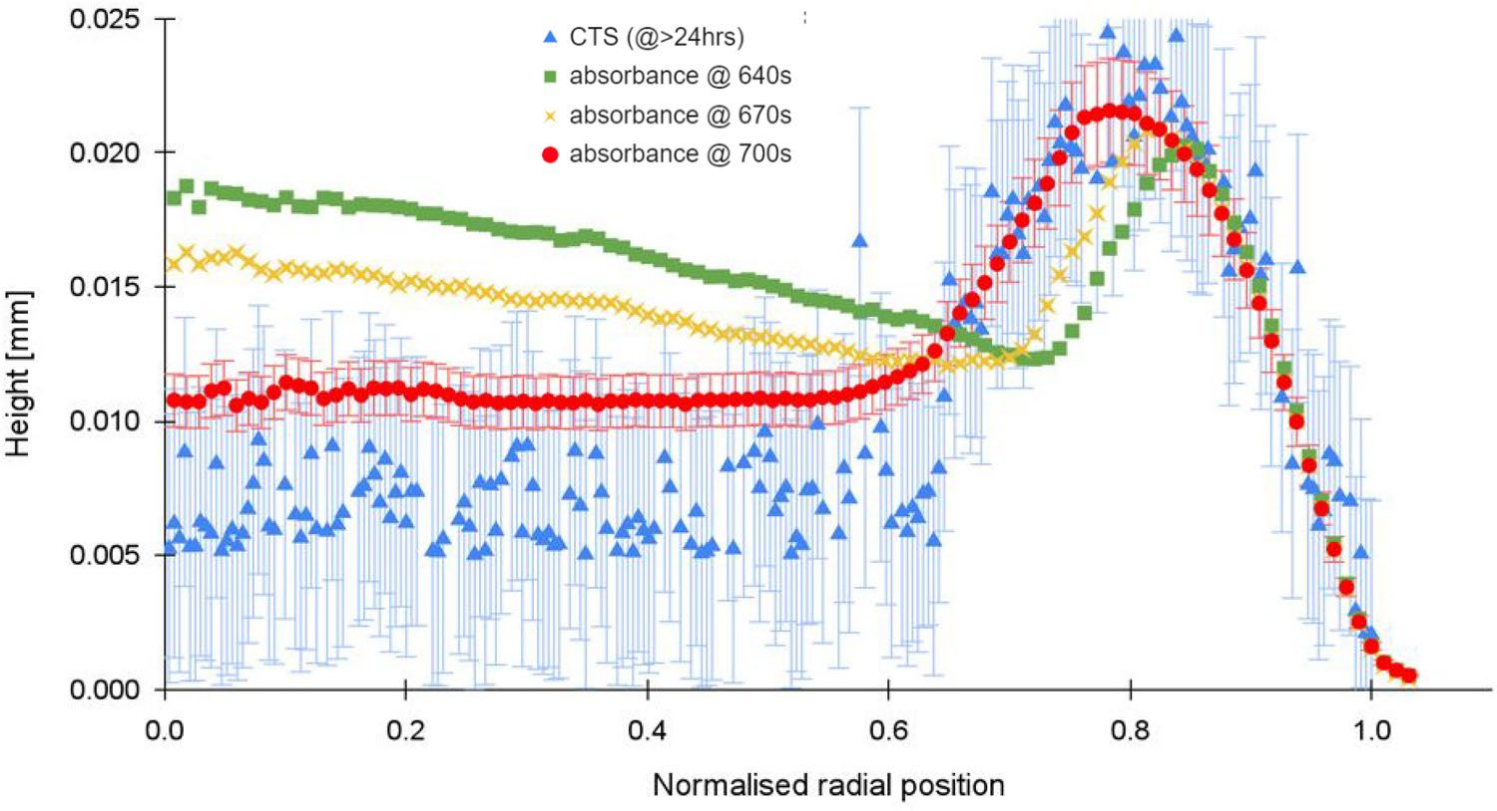
Comparative CTS and absorbance profiles for an evaporated droplet of Polystyrene in H_2_O/D_2_O volume fraction ${\phi }_{0}$ = 15%; *T* = 21 °C; *RH* = 42%. The *t*_*ex*_ measurement of this sample at 700 s also localises the radial peak position, which is coincident with the final residue peak measured using the CTS after 24 h.

Figure 8 shows late-stage evaporation and a final stage CTS and optical absorbance profiles for a sample with initial volume fraction of 15% particulates. Again, the agreement between CTS and optical profile is good, except in the central region. In the late stages of residue formation, scattering and the onset of changes in the extinction coefficient at *t*_*ex*_ may increase the absorbance measurements. The arbitrary timestep may also “miss” the instant of final advection, just before the rapid rise in extinction coefficient exhibited in Fig. 3.

## Conclusions

We adapt a well-known technique for finding the volume fraction of a solution and show its use for eliciting time sequences showing the redistribution patterns of non-volatiles in a drying suspension. The technique has the advantage of simplicity and direct visual corroboration of the shape, velocity and acceleration of the colloidal redistribution packed-front over time.

Whilst retaining the base simplicity of the Beer-Lambert law; we show experimental use of the technique in high volume fraction suspensions, which indicates the technique will be pertinent to tracking a wide range of scenarios; from non-spherical or deformable particulates, non-Newtonian or gelling complex fluids^23,24^.

Implementation is simple and low-cost. Suitable illumination can be chosen by researchers appropriate to a wide range of industrial or biological samples, it allows high volume fractions to be studied and is applicable to a wide range of fluids and suspensions and particle sizes. The technique does not require fluorescence to be introduced. The logarithmic nature of Eq. (1) enables comparison between regions of high and low volume fraction. A suitable calibration image means results can be directly related to final deposit-height measurements. A straightforward method of identifying results distorted by changes in extinction-coefficient has been set out, and the value of its use for time-based studies of advection is described, allowing unphysical results to be excluded.

The technique has the advantage over CTS laser-profiling of being able to resolve movement of suspended material within the liquid, and also over microscopic flow-tracking of the solute front in that the whole geometry of the sample can be observed simultaneously, allowing the velocity of the front to be calculated from time-lapse imaging.

The technique can be applied to all sufficiently-thin scenarios, particular droplet geometries governed by surface tension, including mapping flow and deposition patterns in microfluidic devices^25^, deposition analysis in design and printing of biocompatible inks^26^, and tracking tissue structures and cell movement and affinities in extracellular matrices. There may also be applications in micro-rheological investigations.

## Data Availability

The datasets used and analysed during the current study are available from the corresponding author on reasonable request.
